# An interesting case report of delayed presentation of visual loss from an Ethmoid Mucocoele. Should we offer emergency decompression?

**DOI:** 10.1016/j.ijscr.2021.105744

**Published:** 2021-03-17

**Authors:** Amit Raithatha, Paula Coyle, Rishi Talwar, Andrew Dias

**Affiliations:** Department of ENT, Luton & Dunstable University Hospital NHS Foundation Trust, Luton, UK

**Keywords:** Ethmoid mucocoele, Emergency endoscopic sinus surgery, Optic nerve compression, Case report

## Abstract

Paranasal sinus mucocoeles commonly involve the frontoethmoidal sinuses and can rarely present with vision changes due to expansion and invasion through the orbit. A 50-year-old female presented out of hours with an acute, 16-h history of complete left sided visual loss, on a background of 3 days of visual changes. A left ethmoid mucocoele extending into the left orbital apex causing compression of the optic nerve was diagnosed on imaging. Emergency endoscopic sinus surgery with decompression and marsupialization of the ethmoid mucocoele was performed, which resulted in improvement of vision. This case is unusual due to improvement in vision despite the length of visual loss prior to surgical intervention. This case demonstrates the importance of considering rhinological causes for vision loss, and how critical early identification and surgical intervention can be to prevent serious complications such as permanent vision loss.

## Highlights

• Consider rhinological causes for unilateral vision disturbances or loss.
• Consider emergency flexible nasendoscopy and imaging to identify sinonasal disease.
• Consider emergency endoscopic intervention in compressive Optic neuropathy.
• Communicate frankly with patients about the chances of success and complications.
• Pre-operative bedside assessment of vision is recommended at the minimum.

## 1 Introduction

Acute vision loss can be a transient disturbance (lasting <24 h) or persisting disturbance (lasting >24 h) [1]. The many causes of acute vision loss and the time-sensitive need for evaluation and treatment pose diagnostic and therapeutic challenges [2]. Traditional teaching suggests that irreversible visual loss occurs within 45–120 min after optic nerve injury [3,4]. In acute cases, irreversible visual loss can occur after 120 min, unless the orbital pressure is reduced, thus restoring blood supply and decompressing the optic nerve [5]. Loo et al. and Yoo-Suk et al. highlight the need to treat any presence of infection promptly as part of the management of treating a mucocoele surgically, which may improve optic nerve neuropathy. Failing to treat an infection associated with a mucocoele promptly has a negative influence on post-operative visual outcomes [6,7]. Our case report highlights a rhinological cause for vision loss, and that despite optic nerve compression for a prolonged period of time, with a proactive approach to intervention there can still be some improvement in vision. This work has been reported in line with the SCARE 2020 criteria [8].

## 2 Case presentation

**Fig. 1:**
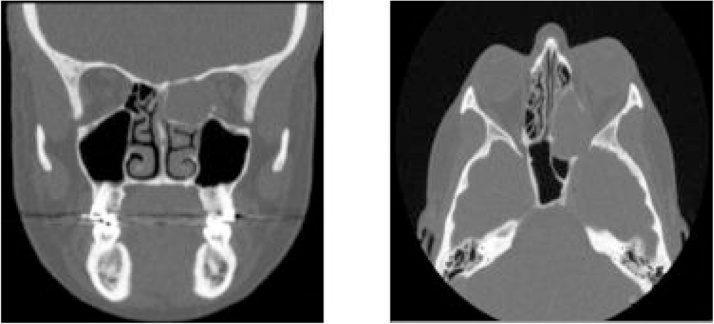
CT Sinuses, coronal view (a) and axial view (b) demonstrating a large, left-sided ethmoid mucocoele extending through the left optic canal and compromising the left optic nerve. CT sinuses: Coronal view (figure a, left) and Axial view (figure b, right) demonstrating a large, left-sided Ethmoid mucocoele extending through the left Optic canal and compressing the left Optic nerve. 127 × 57 mm (150 × 150 DPI).

An otherwise well and fully independent 50-year-old Caucasian female, who reported previously normal visual acuity (6/6 bilaterally), presented with a 16-h history of complete left sided visual loss on a background of blurry vision for the preceding 3 days. When her left eye became blurry, she sought an opinion from her local Ophthalmology department, who could not find a cause. She denied any sinonasal or other symptoms. On examination, she had full, painless eye movements, no proptosis and aside from the visual disturbance in the left eye, there was only a small bruise at the left medial canthus and some mild puffiness of the medial aspect of the left upper eyelid. Flexible nasendoscopy revealed a large septal perforation and bulging left ethmoid bulla without evidence of nasal polyps or discharge. Urgent CT imaging showed a large left ethmoid mucocoele with associated pressure erosion of the medial bony wall of the left optic canal and compromise of the left optic nerve (Fig. 1 a and b). During an in-depth discussion with the patient and considering the total duration of her symptoms, the unlikelihood that surgery would restore her vision was emphasised. The patient consented to emergency endoscopic sinus surgery with decompression of the ethmoid mucocoele. Post-operatively, she was commenced on intravenous ceftriaxone, intravenous dexamethasone, regular nasal douching, 0.1% xylometazoline hydrocholoride and analgesia. Intra-operative swab results subsequently confirmed growth of *Staphylococcus aureus* and directed an antibiotic switch to a 10-day course of intravenous Flucloxacillin.

As immediate pre-operative formal ophthalmology assessment was not available out of hours, the first formal visual assessment was day 1 post-operatively where the visual acuity of her left eye was 6/60 and subjectively the patient self-reported a 50–60% improvement in her vision, particularly in her peripheral vision. Day 3 post-operatively, she underwent repeat CT imaging. This showed a small bony fragment and some residual ethmoidal disease, which required revision FESS and orbital decompression jointly with the specialist Rhinologist. This resulted in further improvement of the patient's visual acuity to 6/36 by the next day.

## 3 Follow up & outcomes

Four months later she was discharged from ophthalmology follow-up with a stable visual acuity of 6/36 in the left eye. 6/36 means that she can see an object that is 6 feet away clearly, whereas somebody with normal vision could clearly see this object from 36 feet away. As the vision in her right eye was normal, this caused her to have distorted depth perception, which she was advised would further improve over the next 3–4 months as she adapted.

## 4 Discussion

Ethmoid sinuses are paired sinuses that vary in size and number. They lie between the nasal septum and orbit divided by the thin lamina papyracea. Permanent obstruction of a sinus ostium leads to the trapping and accumulation of sinus secretions [9]. A mucocoele is a cystic lesion of the epithelial layer that lines the paranasal sinuses and contains thick mucus inside. A mucocoele expands gradually and exerts pressure erosion on the sinus walls, leading to displacement of adjacent structures. Mucocoeles can develop in any sinus but the commonest site is the frontal followed by the ethmoid [10]. Mucocoeles can be caused by trauma, previous surgery, infection, or inflammation, but can also be idiopathic. Paranasal sinus mucocoeles can induce headache, periorbital pain and visual disturbance. Frontal and ethmoidal mucocoeles usually present with ophthalmic symptoms such as proptosis, diplopia, reduced range of eye movement and eyelid swelling. Visual impairment can be due to compression with vascular compromise, spread of inflammation or infection to the optic nerve [11]. Treatment consists of decompression surgery, which should be performed as an emergency if there are associated visual symptoms. Recently, the endoscopic approach has become preferred over the external approach because of minimal mucosal injury, shorter recovery time and low recurrence rate [[12], [13], [14]]. Guidelines for surgery via either approach is to identify the skull base posteriorly, marsupialise widely and remove all osteitic bone from the opening so as to make it flush with the surrounding bone to reduce the chance of recurrence. This case emphasises that rhinological causes should be considered and shows that a proactive approach can improve symptoms significantly even at a late stage, thus suggesting that compressive optic neuropathy may benefit from surgical intervention even if significant visual loss has been sustained many hours earlier. Optical coherence tomography (OCT) has been used to predict the prognosis if compressive optic neuropathies, but there is no published evidence specifically with mucocoeles [15].

## 5 Learning points & conclusions

- Always consider rhinological causes for unilateral acute vision loss.
- Emergency flexible nasendoscopy and imaging is required to rule out sinonasal pathology. CT provides anatomical detail and bony delineation to plan surgery, but MRI is superior for demonstrating soft tissue relationships and differentials.
- Emergency surgical intervention should be considered as soon as possible in Compressive Optic Neuropathy, even if visual loss has been sustained for longer than 2 h, to prevent further visual compromise and spreading infection.
- The endoscopic approach is preferred to the open approach as there is less risk of mucosal injury.
- In emergency cases, have frank conversations with patients regarding the chances of surgical success and potential complications.
- In emergency cases, do not wait for formal Ophthalmology assessment if it is not immediately available, but we suggest performing a non-specialist bedside clinical assessment of vision, e.g. Using a Snellen chart, testing visual fields, examining the fundi with an Ophthalmoscope, assessing colour vision and for relative afferent pupillary defect. Equipment needed to make these assessments should be available in most A&E departments [16].

## 6 Patient perspective

A discussion with the patient after discharge revealed that for the preceding couple of years she suffered with discomfort in her head when running on hard surfaces. Fortunately, she has noticed that since the surgical intervention this has never happened again. She was appreciative of receiving prompt out of hours, emergency intervention and subjectively reported some gradual improvement in her vision in regard to depth perception post-discharge over time.

## Declaration of Competing Interest

There were no conflicts of interest during the writing or submission of this article.

## Sources of funding

No funding was required.

## Ethical approval

Ethical approval was not sought for this case report as it was not required due to the nature of the case report.

## Consent

Written informed consent was obtained from the patient for publication of this case report and accompanying images. A copy of the written consent is available for review by the Editor-in-Chief of this journal on request.

## Author contribution

All authors were involved in the care of this patient and made substantial contributions. Additionally, Amit Raithatha was involved in the writing of the draft manuscript, which was reviewed and edited by Paula Coyle and Rishi Talwar.

## Registration of research studies

Not applicable.

## Guarantor

Amit Raithatha.

## Provenance and peer review

Not commissioned, externally peer-reviewed.

## References

[1] Bagheri N., Mehta S. (2015). Acute vision loss. Prim. Care.

[2] Borooah S., Dhillon A., Dhillon B. (2015). Gradual loss of vision in adults. BMJ.

[3] Lafuente M.P., Villegas-Perez M.P., Selles-Navarro I., Mayor-Torroglosa S., Mirallesde Imperial J., Vidal-Sanz M. (2002). Retinal ganglion cell death after acute retinal ischemia is an ongoing process whose severity and duration depends on the duration of the insult. Neuroscience.

[4] Adachi M., Takahashi K., Nishikawa M., Miki H., Uyama M. (2021). High intraocular pressure induced ischemia and reperfusion injury in the optic nerve and retina in rats. Graefes Arch. Clin. Exp. Ophthalmol..

[5] Cook C. (2018). Emergency management: optic nerve compression. Community Eye Health.

[6] Loo (2009). Visual outcomes in patients with paranasal mucoceles. Ophthal. Plast. Reconstr. Surg..

[7] Yoo-Suk K. (2011). Paranasal sinus mucoceles with ophthalmologic manifestations: a 17-year review of 96 cases. Am. J. Rhinol. Allergy.

[8] Agha R.A., Franchi T., Sohrabi C., Mathew G., for the SCARE Group (2020). The SCARE 2020 Guideline: updating consensus surgical CAse REport (SCARE) guidelines. Int. J. Surg..

[9] Capra G.G., Carbone P.N., Mullin D.P. (2012). Paranasal sinus mucocele. Head Neck Pathol..

[10] Morita S., Mizoguchi K., Iizuka K. (2010). Paranasal sinus mucoceles with visual disturbance. Auris Nasus Larynx.

[11] Chiarini L., Nocini P., Bedogni A., Consolo U., Giannetti L., Merli G. (2000). Intracranial spread of a giant frontal mucocele: case report. Br. J. Oral Maxillofac. Surg..

[12] Mora-Horna E.R., Lopez V.G., Anaya-Alaminos R. (2015). Optic neuropathy secondary to a sphenoid ethmoidal mucocele: case report. Arch. Soc. Eps Oftalmol..

[13] Bockmuhl U., Kratzsch B., Brenda K., Draf W. (2005). Paranasal sinus mucoceles: surgical management and long-term results. Laryngorhinotologie.

[14] Lund V.J. (1998). Endoscopic management of paranasal sinus mucocoeles. J. Laryngol. Otol..

[15] Micieli J.A., Newman N.J., Biousse V. (2019). The role of optical coherence tomography in the evaluation of compressive optic neuropathies. Curr. Opin. Neurol..

[16] Corbett J. (2003). The bedside and office neuro-ophthalmology examination. Semin. Neurol..

